# Silane-catalysed fast growth of large single-crystalline graphene on hexagonal boron nitride

**DOI:** 10.1038/ncomms7499

**Published:** 2015-03-11

**Authors:** Shujie Tang, Haomin Wang, Hui Shan Wang, Qiujuan Sun, Xiuyun Zhang, Chunxiao Cong, Hong Xie, Xiaoyu Liu, Xiaohao Zhou, Fuqiang Huang, Xiaoshuang Chen, Ting Yu, Feng Ding, Xiaoming Xie, Mianheng Jiang

**Affiliations:** 1 State Key Laboratory of Functional Materials for Informatics, Shanghai Institute of Microsystem and Information Technology, Chinese Academy of Sciences, 865 Changning Road, Shanghai 200050, China; 2 Graduate University of the Chinese Academy of Sciences, Beijing 100049, China; 3 School of Physics and Electronics, Central South University, Changsha 410083, China; 4 Institute of Textiles and Clothing, Hong Kong Polytechnic University, Kowloon, Hong Kong 999077, China; 5 Division of Physics and Applied Physics, School of Physical and Mathematical Sciences, Nanyang Technological University, 21 Nanyang Link, Singapore 637371, Singapore; 6 National Laboratory for Infrared Physics, Shanghai Institute of Technical Physics, Chinese Academy of Sciences, Shanghai 200083, China; 7 CAS Key Laboratory of Materials for Energy Conversion, Shanghai Institute of Ceramics, Chinese Academy of Sciences, Shanghai 200050, China; 8 School of Physical Science and Technology, ShanghaiTech University, 319 Yueyang Road, Shanghai 200031, China

## Abstract

The direct growth of high-quality, large single-crystalline domains of graphene on a dielectric substrate is of vital importance for applications in electronics and optoelectronics. Traditionally, graphene domains grown on dielectrics are typically only ~1 μm with a growth rate of ~1 nm min^−1^ or less, the main reason is the lack of a catalyst. Here we show that silane, serving as a gaseous catalyst, is able to boost the graphene growth rate to ~1 μm min^−1^, thereby promoting graphene domains up to 20 μm in size to be synthesized via chemical vapour deposition (CVD) on hexagonal boron nitride (*h*-BN). Hall measurements show that the mobility of the sample reaches 20,000 cm^2^ V^−1^ s^−1^ at room temperature, which is among the best for CVD-grown graphene. Combining the advantages of both catalytic CVD and the ultra-flat dielectric substrate, gaseous catalyst-assisted CVD paves the way for synthesizing high-quality graphene for device applications while avoiding the transfer process.

## Abstract (web summary)

The growth of high-quality graphene directly on to dielectric substrates is of key importance for future electronic and optoelectronic applications. Here, the authors use silane-catalysed chemical vapour deposition to fabricate large graphene sheets, up to 20 μm in size, on hexagonal boron nitride.

## Graphical abstract

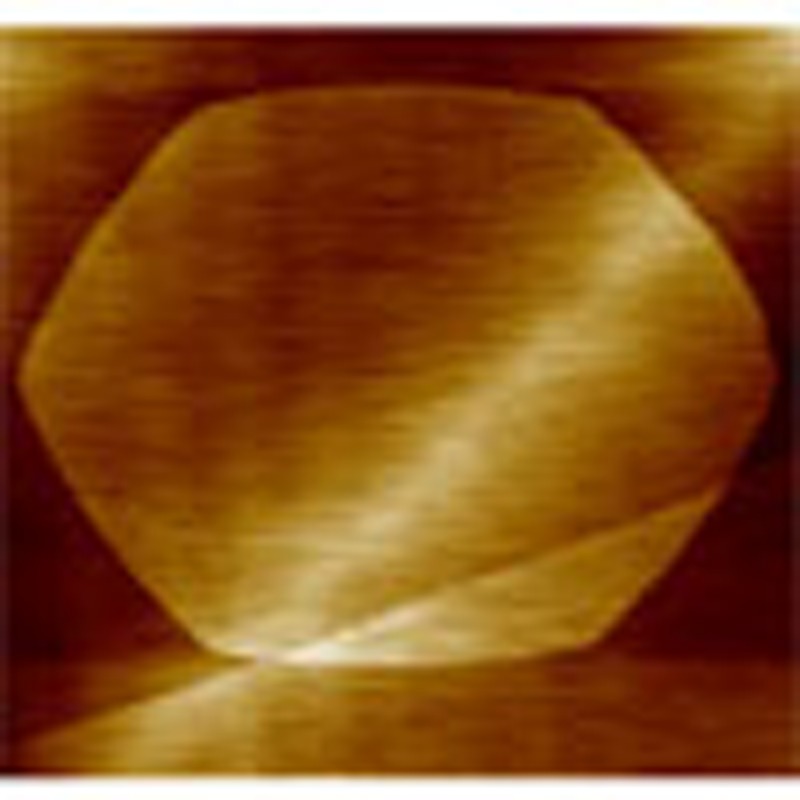

As the first isolated atomically thin crystal^1^, graphene has attracted enormous interest from people all over the world because of its rich physical properties and great potential in various applications. Among them, applications in electronics are the most appealing, although they are challenging because they require high-quality, large-area samples^2^. Among the popular methods of graphene synthesis, chemical vapour deposition (CVD) is known as the most promising for scalable growth of high-quality graphene sheets^3^,^4^,^5^,^6^,^7^,^8^,^9^,^10^,^11^,^12^,^13^,^14^,^15^,^16^,^17^,^18^. Progress in this area, as demonstrated by the successful synthesis of 30-inch continuous films^19^, the wafer-scale growth of a single-crystal monolayer of graphene on Ge^20^, and centimetre-sized graphene domains^15^, has been achieved. However, post-growth transfer remains a major hurdle for further applications because transfer processes introduce unavoidable surface/interface contamination, cracks and excessive wrinkles in graphene. Moreover, owing to the rough catalytic metal surface, the quality of the graphene grown is still far from the flatness of the mechanically exfoliated samples. Thus, the ability to develop a transfer-free technique by growing graphene directly on dielectric substrates with a large domain size, fast growth rate and very high quality is highly desirable.

Recently, molybdenum disulfide, tungsten disulfide and hexagonal boron nitride *(h*-BN) are found to be good candidates for the substrate of graphene devices^21^. Among them, *h*-BN is obviously the best one because of its insulation properties, chemical inertness and small lattice misfit. Growing graphene directly on ultra-flat *h*-BN can greatly preserve the pristine properties of graphene, and the mobility of the aligned graphene grown on *h*-BN via the CVD method reaches 20,000–30,000 cm^2^ V^−1^ s^−1^ at room temperature^22^. However, this growth method suffers from the great drawback of very low growth rate (~1 nm min^−1^) and the generation of domain sizes that are mostly smaller than 1 μm. Although the fabrication of a graphene domain 11 μm in size has been achieved, it took 72 h to complete the fabrication^23^. Such a slow growth rate, which is three to four orders of magnitude slower than that obtained by CVD on a catalytic metal surface, originates mainly from the lack of a catalyst in the process.

In this report, we demonstrate that silane and germane as gaseous catalysts can boost the growth rate of graphene on *h*-BN by almost two-order of magnitude higher than that in the absence of gaseous catalyst. The introduction of the gaseous catalyst also effectively improves the percentage of precisely aligned domains on *h*-BN. These results present a promising route towards very high-quality, transfer-free graphene on dielectric substrates for electronic applications.

## Results

### Gaseous catalyst assisted (GCA) graphene growth

**Figure 1:**
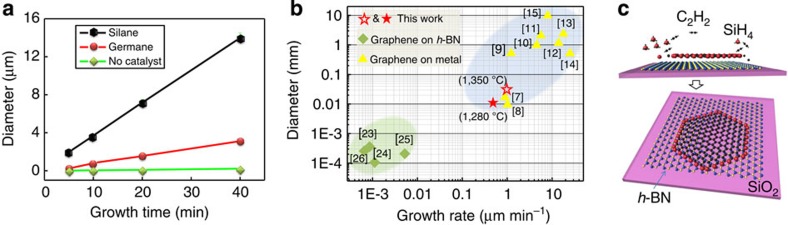
The gaseous catalyst-assisted CVD of graphene on *h*-BN. (**a**) The growth duration dependence of the domain dimensions for single-crystalline graphene in the presence of silane (black) or germane (red) gaseous catalysts and no catalyst (green). The diameter of the graphene domain is measured as the diagonal length of the hexagonal graphene crystal, and the growth temperature is 1,280 °C. (**b**) The graphene growth rates plotted as a function of the dimensions of the single-crystalline domains obtained in this work and those reported in the literature. The data points are labelled with the reference number from whence they came in brackets. (**c**) Schematic of the gaseous catalyst-assisted chemical vapour deposition process, visualizing the carbon (black), nitrogen (blue), boron (yellow), silicon (red) and hydrogen (small grey) atoms as spheres in the illustration. The schematic illustrates simplified schemes of the catalytic growth of monolayer graphene onto *h*-BN, where silicon atoms from the decomposition of SiH_4_ attach to the edge of the graphene and assist its growth.

A series of experiments are carried out to understand the effects of different gaseous catalysts upon the growth of graphene. Figure 1a presents the dependence of the domain size upon the growth time at a growth temperature of 1,280 °C. It is found that the domain size always increases linearly with growth time. Also, it is found that the growth rate of graphene in the absence of a catalyst is about 5 nm min^−1^, which increases by an order of magnitude to 50 nm min^−1^ when germane is introduced. If silane is introduced, the growth rate further increases to 400 nm min^−1^ and, if the substrate is further heated to 1,350 °C, the growth rate reaches ~1,000 nm min^−1^, which is very close to that of graphene grown on a metal surface by CVD (Fig. 1b). At such a high growth rate, the largest domain size of the graphene grown on *h*-BN reaches 20 μm after 20 min of growth (Fig. 1b). As shown in Fig. 1b^7^,^8^,^9^,^10^,^11^,^12^,^13^,^14^,^15^,^24^,^25^,^26^,^27^, using silane as a catalyst improves both the graphene growth rate and the typical domain size on *h*-BN by two orders of magnitude when compared with no catalyst. Auger electron spectroscopy is used to evaluate the amount of Si and Ge residue in the grown graphene samples (see Supplementary Fig. 4), whereupon no Si or Ge signal is detected, confirming that silane and germane are suitable to serve as catalysts for graphene growth. It is worthwhile to note that excessive supply of silane will cause formation of SiC nanoparticles (see Supplementary Fig. 3). The GCA-CVD method thereby bridges the huge gap between the direct growth of graphene on a dielectric substrate and that on a metal surface, and the graphene growth on *h*-BN presented here has already entered into the practical application regime. Figure 1c schematically illustrates the mechanism of graphene growth on *h*-BN, where the silicon atoms attach to the edge of the graphene domain and serve as the catalyst to reduce the reaction barrier for C_2_H_2_ molecules to form the honeycomb lattice along the graphene edge during growth.

### Simulation of growth mechanism

**Figure 2:**
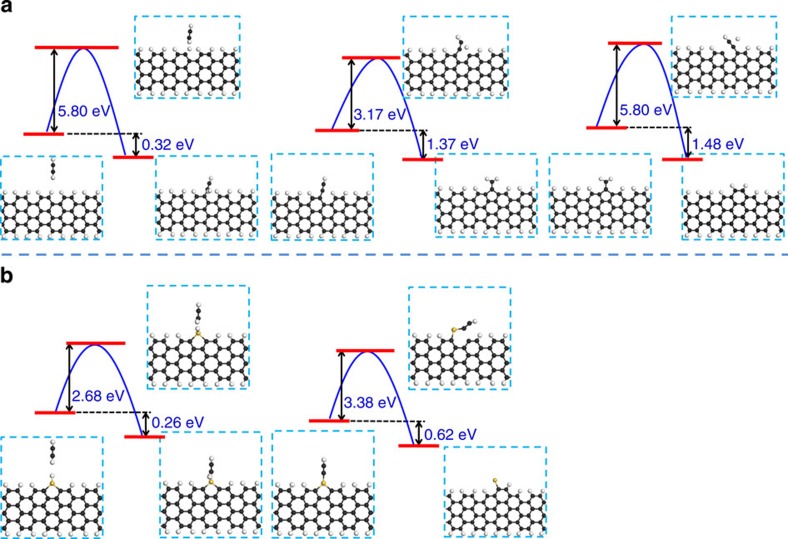
Density functional calculation of the reaction between a C_2_H_2_ molecule with the commonly observed armchair graphene edge. (**a**) without and (**b**) with an Si atom as a catalyst on the graphene edge. The black, white and yellow balls represent carbon, hydrogen and silicon atoms, respectively.

To achieve a more detailed understanding of the role of silicon in graphene CVD growth, density functional theory (DFT) calculations are performed on armchair graphene, with the results shown in Fig. 2. Without a catalytic metal surface, the graphene growth front must be passivated by H atoms as the reaction is carried out in a hydrogen-rich environment. The chosen carbon feedstock, C_2_H_2_, ensures the formation of a complete hexagon for each cycle of C_2_H_2_ incorporation at the graphene edge. Our calculation indicates that three reaction steps are required to incorporate a C_2_H_2_ molecule onto the graphene edge to form a new hexagon, with corresponding energy barriers for these reaction steps of 5.80, 3.17 and 5.80 eV. In the case of a silicon catalyst (Fig. 2b), when a silicon atom attaches to the growth front, only two steps are required to incorporate a C_2_H_2_ onto the graphene edge to form a new hexagon with corresponding energy barriers of 2.68 and 3.38 eV. Because each reaction step is exothermic, the threshold barriers for C_2_H_2_ incorporation onto a graphene edge without and with a Si atom as a catalyst are 5.80 and 3.38 eV, respectively. These calculations clearly indicate that this much-reduced threshold barrier when a catalyst is involved is responsible for the increased growth rate. Graphene edge activation by metal ad-atoms has been studied previously by simulations^28^,^29^,^30^,^31^. Experimentally, copper vapour was used in the demonstration of graphene growth on insulating substrates^32^,^33^. Oxygen was another surface activator, accelerating the graphene growth on Cu and suppressing its nucleation at the same time^15^. The use of silane/germane, conventional gases used in semiconductor industry with good accessibility and controllability, has not been previously proposed.

### Atomic force microscopy characterization and alignment survey

**Figure 3:**
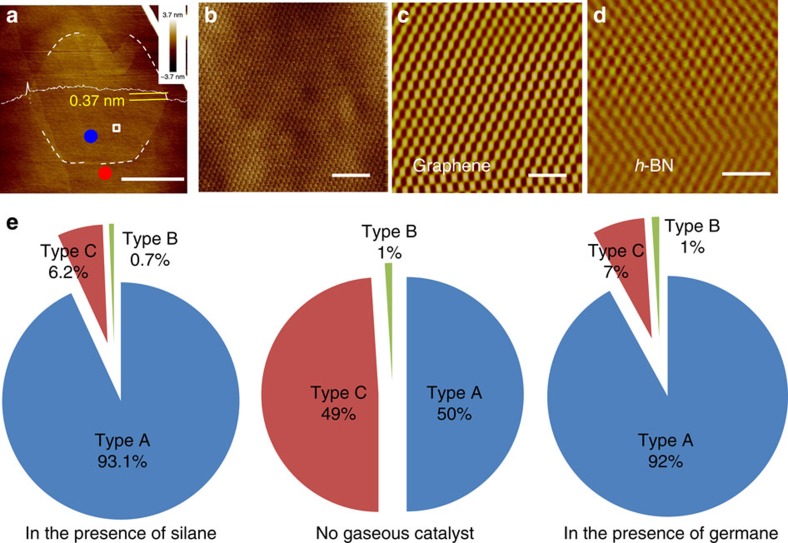
The investigation on crystallinity and alignment of graphene domains grown on *h*-BN. (**a**) Topography image of a typical single-crystalline graphene domain with a diameter of 20 μm. The dashed line frames the shape of the graphene grain. The line scan shows the graphene thickness to be 0.37 nm. The scale bar is 10 μm. (**b**) The AFM friction image of the selected area in **a**, where the giant moiré pattern with a periodicity of 13.9 nm can be clearly seen. The scale bar is 100 nm. Atomic-resolution AFM images of (**c**) graphene and (**d**) *h*-BN taken from the areas marked by the blue and red dots in **a**, respectively. During the measurement, the scanning angles are always kept the same. The scale bars are 1 nm. (**e**) Pie charts of the distribution of the type of graphene domains obtained with/without gaseous catalysts. Type ‘A’ indicates a graphene domain that is precisely aligned with the underlying *h*-BN, type ‘B’ is one whose lattice is rotated 30° with respect to the underlying *h*-BN lattice, and type ‘C’ is one with a polycrystalline structure.

The crystallinity and alignment of the graphene domains can be studied by analysing the moiré patterns obtained by atomic force microscopy (AFM), where the integrity of the pattern gives information of graphene crystallinity and the alignment can be judged by the periodicity and the rotation of the moiré pattern with respect to the *h*-BN lattice^22^. M. Yankowitz *et al*. also did systematic investigations of the graphene/*h*-BN super-lattice with different angular rotation^34^. In our experiments, three types of domains are observed. The ‘A’ domains present a regular hexagonal shape with a giant moiré pattern, whose edges are along the armchair direction. The periodicity of the moiré pattern is about 13.9 nm, which demonstrates that these graphene domains are precisely aligned with the underlying *h*-BN^22^. The ‘B’ domains also exhibit a regular hexagonal shape but with no detectable moiré patterns, and detailed atomic-resolution AFM measurements indicate that all of these ‘B’ graphene domains are rotated ~30° with respect to the underlying *h*-BN lattice. The ‘C’ domains exhibit the typical polycrystalline structure, with a detectable moiré pattern only on some sub-domains. Figure 3a shows a typical ‘A’ domain with a giant moiré pattern, shown in Fig. 3b, with the atomic-resolution image of the graphene and the underlying *h*-BN shown in Fig. 3c,d, respectively. The alignment of the moiré pattern with respect to the *h*-BN is very sensitive to the angular separation between the graphene and *h*-BN when the angular separation is less than 1° (ref. 22). Although the measurement may possess **±3° measurement error for the lattice orientation, mis-orientation between the graphene and *h*-BN should be less than 0.05° by evaluating the alignment of the moiré pattern with respect to the *h*-BN. These results prove that the graphene is precisely aligned with the underlying *h*-BN. Details about the other two types of domains are given in Supplementary Figs 6 and 7 and Supplementary Discussion.

Figure 3e shows the statistics of the types of graphene domains grown with and without gaseous catalysts. In the absence of a gaseous catalyst, the precisely aligned domains (Type A) and polycrystalline domains (Type C) appear in almost equal probability and only ~1% domains are the 30°-rotated single-crystalline domain (Type B). When using silane or germane as a catalyst, however, the percentage of precisely aligned domains (Type A) greatly improves to 93.1 or 92%.

### Raman characterization of graphene domains grown on *h*-BN

**Figure 4:**
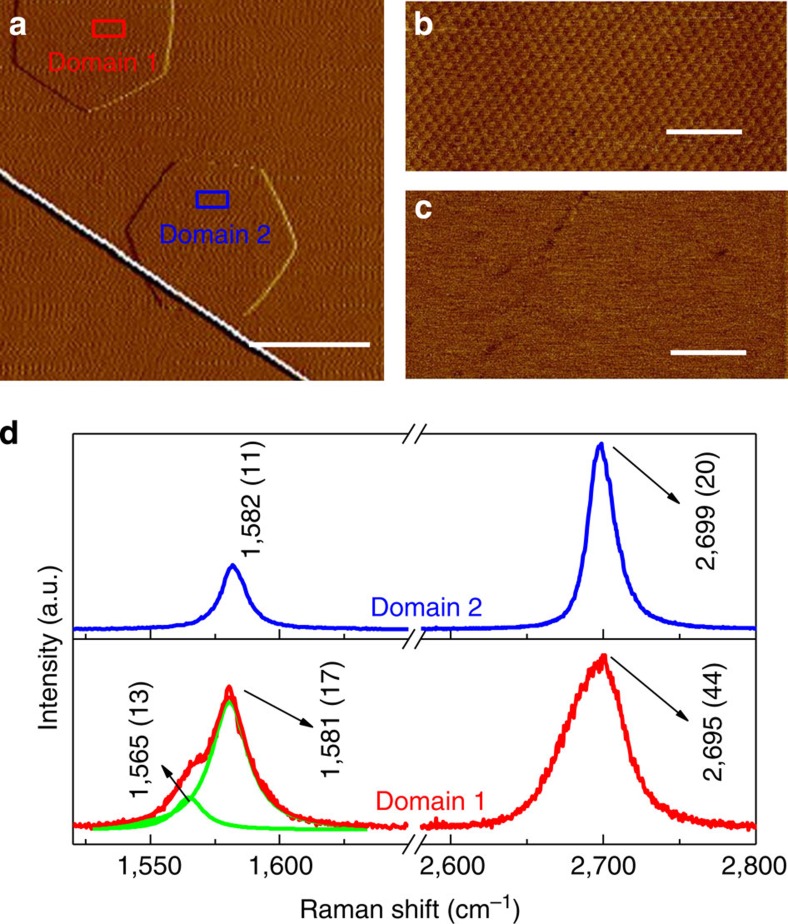
Raman analyses on graphene domains grown on *h*-BN. (**a**) Topography images of two graphene domains grown on an *h*-BN surface. The white line across the image indicates a wrinkle on the *h*-BN surface formed during cooling. The scale bar is 2 μm. (**b**) Magnified view from the red box in **a**, where the presence of a giant moiré pattern indicates precisely aligned graphene with respect to the *h*-BN. (**c**) Magnified view from the blue box in **a**, where no detectable moiré pattern is seen. The scale bars in **b** and **c** are 100 nm. (**d**) Raman spectra taken from Domain 1 (lower plot) and Domain 2 (upper plot) in **a**. The full-width at half-maximum for each peak is given in parentheses with the peak location value, and the wavelength of the exciting laser is 488 nm.

Figure 4 shows the Raman spectra from Domain 1 (which is precisely aligned and noted as ‘type A’) and Domain 2 (type ‘B’, which is a 30°-rotated single-crystalline domain). The Raman results for the aligned or misaligned graphene domains grown on *h*-BN by the GCA-CVD method are very similar to those for aligned or misaligned graphene flakes on *h*-BN made by mechanical exfoliation^35^, indicating that the GCA-CVD-grown graphene domains are of very high quality. It can be observed that the Raman spectrum of precisely aligned graphene exhibits obvious differences from that of rotated domains. In the precisely aligned graphene, the full-width at half-maximum (FWHM) of the 2D band is ~44 cm^−1^, which is broader than that of graphene domains with a large misalignment angle. Moreover, a shoulder peak located at 1,565 cm^−1^ that is non-dispersive (see Supplementary Fig. 8) is noticeable, which can be attributed to a transverse optical phonon and may be activated by folding^35^,^36^. In addition, a more comprehensive analysis of the Raman results of the GCA-CVD-grown graphene domains is given in Supplementary Fig. 8 and Supplementary Table 2.

### Electronic transport properties

**Figure 5:**
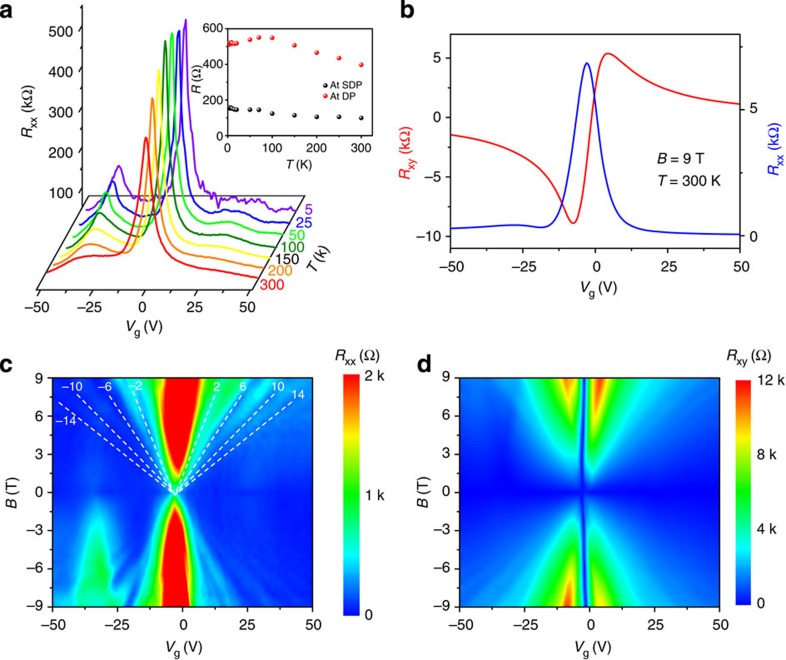
Transport measurement of the single-crystalline graphene precisely aligned with the underlying *h*-BN. (**a**) Back-gate voltage (*V*_g_) dependence of the longitudinal resistance at different temperatures. Inset shows the temperature dependence of the resistance at the Dirac point (DP; red spheres) and satellite peaks at the hole doping Secondary Dirac point (SDP) (black spheres). (**b**) Longitudinal (*R*_xx_, blue) and Hall resistance (*R*_xy_, red) versus *V*_g_ at temperature *T*=300 K and magnetic field *B*=9 T. (**c**,**d**) Quantum Hall effect fan diagram of (**c**) *R*_xx_ and (**d**) *R*_xy_ as a function of *V*_g_ and *B* at a temperature of 2 K.

A Hall bar device is made on a heavily doped silicon substrate for measuring the Hall and field effect mobilities (for details of the device fabrication, see Methods and Supplementary Fig. 9). Figure 5 shows the field effect characteristics of the device (Fig. 5a), the results of the Hall measurements at a temperature of 300 K and a magnetic field of 9 T (Fig. 5b), and the effects of Landau level splitting upon the longitudinal and Hall resistances at a temperature of 2 K (Fig 5c–d). The carrier-independent field effect mobility can be calculated from the plot in Fig. 5a, and is found to be 17,000 cm^2^ V^−1^ s^−1^ at 300 K. The hole and electron mobilities can be derived from Fig. 5b, with values of 19,000 and 23,000 cm^2^ V^−1^ s^−1^, respectively. The high-mobility value measured for these samples indicates that the electrical quality of the graphene domains on *h*-BN is among the best for CVD-grown graphene^15^,^37^,^38^ and is comparable to that of micromechanically exfoliated graphene^39^ (see Supplementary Table 3).

Two side peaks are observed in Fig. 5a owing to secondary Dirac cones, which are a result of the graphene/*h*-BN moiré pattern, and are consistent with other reports^40^,^41^,^42^. It is worth noting that the resistances at the Dirac Point do not significantly increase with decreasing temperatures. Woods *et al*. have reported similar results, which in their study were explained as a result of the suppression of the commensurate state^43^.

In the plots of *R*_xx_ and *R*_xy_ as a function of both gate voltage and magnetic field, the standard Quantum Hall effect for graphene is observed to exhibit valleys in *R*_xx_ (Fig. 5c) and plateaus in *R*_xy_ (Fig. 5d) at the filling factors *ν*=±4(*n*+1/2)=±2,±6,±10…, where *n*=0, 1, 2… is the Landau level index. A further investigation to demonstrate the effects of the secondary Dirac point upon the magnetic field is still underway.

## Discussion

In summary, by combining the advantages of the catalytic CVD method and an ultra-flat dielectric substrate, we have developed a novel method to synthesize graphene domain sizes up to 20 μm on *h*-BN with a growth rate comparable to that of graphene growth on metal surfaces and a graphene quality comparable to that made by the mechanical exfoliation method. In addition, the synthesized graphene on *h*-BN is transfer-free, which allows further electronic applications based on graphene/dielectric hetero-structures. Most of the graphene domains are perfectly aligned with the *h*-BN. No grain boundaries are observed when two domains merge; this brings hope to future fabrication of single crystalline graphene/h-BN wafer, which is essential for its scalable application on electronics.

## Methods

### Graphene synthesis

Before graphene growth, *h*-BN flakes were mechanically exfoliated onto quartz by Nitto tape and, after acetone cleaning, the quartz substrate with the *h*-BN flakes was loaded into the growth system. The graphene growth was carried out in a low-pressure CVD furnace at a temperature of 1,100–1,400 °C. The system was then heated to 1,280 °C under an Ar/H_2_ (5:1) mixture flow of 10 cm^3^ per min (s.c.c.m.), corresponding to 15 Pa, and annealed for 5 min, after which the Ar/H_2_ flow was turned off. The C_2_H_2_ flow and a mixture of silane/argon or germane/argon (5% mole ratio of silane or germane to argon) were introduced into the system for the graphene growth. The pressure was kept at 5 Pa during the growth, and the growth time was in the range of 5–40 min. After growth, both the C_2_H_2_ and gaseous catalyst flow were turned off and the system was cooled down to room temperature with the Ar/H_2_ mixture flowing. The samples of graphene on *h*-BN grown on a quartz surface were first transferred to a highly doped p-type silicon wafer with a 300-nm-thick SiO_2_ capping layer for electrical transport studies.

### Scanning probe microscopy

As-grown samples were characterized by an AFM (Dimension Icon, Bruker) in contact mode, and the atomic-resolution images were obtained by the AFM (Multimode III, Veeco) under ambient conditions. The AFM images on different surfaces were recorded in contact mode using SNL-10 AFM tips from Bruker that possess a nominal tip radius of less than 10 nm, and whose cantilevers possess a force constant *k*=0.05–0.5 N m^−1^. To obtain a high accuracy, scanners with a travel range less than 10 μm along the *X* and *Y* directions were used. Calibration for the atomic resolution was performed with newly cleaved highly ordered pyrolytic graphite (HOPG) before the measurements where, after calibration, the mean distance between the vicinal carbon atoms was measured to be 0.142 nm. The integral gain and set-point were adjusted to be as low as possible to obtain optimal images, and the scan rate was set to a value in the range of 10–60 Hz to reduce the noise from thermal drift. Several hours of pre-scanning were carried out to warm up the scanner and ensure high imaging stability. Error in this orientation measurement varies by data points and was typically ±2°, although it sometimes reached ±3°.

### DFT calculation

All of the calculations were performed within the framework of the DFT as implemented in the Vienna Ab-initio Simulation Package. The exchange-correlation potentials were treated by the gradient density approximation, and the interaction between valence electrons and ion cores was described by the projected augmented wave method. The energy cutoff for the plane wave functions was 400 eV and the force acting on each atom was less than 0.02 eV Å^−1^. The energy barrier of every advance step was explored by climbing nudged elastic band method. The vacuum layer inside the super-cell was kept as large as 14 Å to avoid interaction with the adjacent unit cell. The Brillion zone was sampled as 1 × 2 × 1 grid meshes for the 7 × 5 slab and 1 × 1 × 1 grid meshes for the larger 8 × 6 and 7 × 8 slabs using the Monkhorst-Pack scheme during the calculation.

### Raman spectroscopy and mapping

Raman spectra were obtained with a WITec micro-Raman instrument possessing excitation laser lines of 488/532/633 nm. An objective lens of × 100 magnification and a 0.95 numerical aperture was used, producing a laser spot that was ~0.5 μm in diameter. The laser power was kept less than 1 mW on the sample surface to avoid laser-induced heating. The Raman images were acquired using a WITec Raman system with a 600 lines/mm grating and a piezo crystal-controlled scanning stage under a 488-nm laser excitation. The scanning step was about 200 nm.

### Device fabrication and electronic transport measurements

The Hall bar structure of the graphene devices was defined by a standard electron beam lithographic technique. The metal contacts (60 nm Au/10 nm Ti) were deposited through electron beam evaporation, whereupon the devices were annealed in a hydrogen atmosphere at 250 °C for 3 h to remove resist residues and to reduce the contact resistance between the graphene and electrodes before the electrical measurements. Because the thickness of the *h*-BN flake on the 300-nm-thick SiO_2_/Si substrates is about 20 nm, the effective capacitance, *C*_g_, can be estimated as 10.5 nF cm^−2^. Both the electrical transport and magneto-transport measurements were carried out in a physical property measurement system (PPMS from Quantum Design, Inc.) via the standard lock-in technique.

## Author contributions

M.J. and X.X. directed the research work. H.W., X.X. and S.T. conceived and designed the experiments. S.T. fabricated the graphene samples. S.T. and H.W. performed the AFM experiments. H.W., H.X. and X.L. fabricated the electronic devices. H.W., H.S.W. and Q.S. performed the transport measurements. F.D., X.Y.Z., X.C. and X.H.Z. performed the DFT calculations. C.C. and T.Y. performed the Raman measurements. H.W., X.X., S.T., F.D., C.C. and T.Y. analysed the experimental data and designed the figures. H.W., S.T., C.C., T.Y., F.D., F.H., X.X. and M.J. co-wrote the manuscript and all authors contributed to critical discussions of the manuscript.

## Additional information

**How to cite this article**: Tang, S. *et al*. Silane-catalysed fast growth of large single-crystalline graphene on hexagonal boron nitride. *Nat. Commun*. 6:6499 doi: 10.1038/ncomms7499 (2015).

## Supplementary Material

Supplementary Information Supplementary Figures 1-11, Supplementary Tables 1-3, Supplementary Notes 1-3, Supplementary Discussion and Supplementary References
