# Supplementary material for: Silane-catalysed fast growth of large single-crystalline graphene on hexagonal boron nitride
Source: Nat Commun. 2015 Mar 11;6:6499. doi: 10.1038/ncomms7499 (PMC4382696; doi:10.1038/ncomms7499)
Supplement: Supplementary Information — Supplementary Figures 1-11, Supplementary Tables 1-3, Supplementary Notes 1-3, Supplementary Discussion and Supplementary References [file ncomms7499-s1.pdf]

Supplementary Figures

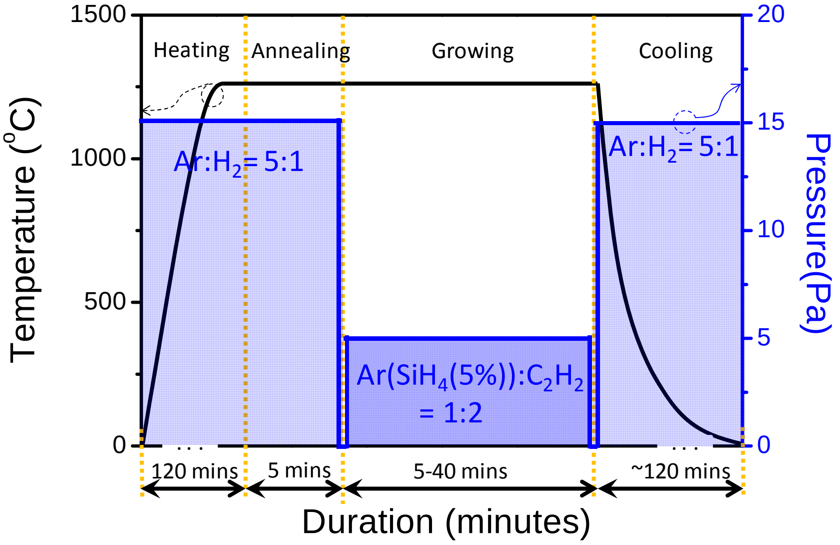

Supplementary Figure 1 | Schematic diagram of a typical process for graphene growth on *h*-BN by GCA-CVD.

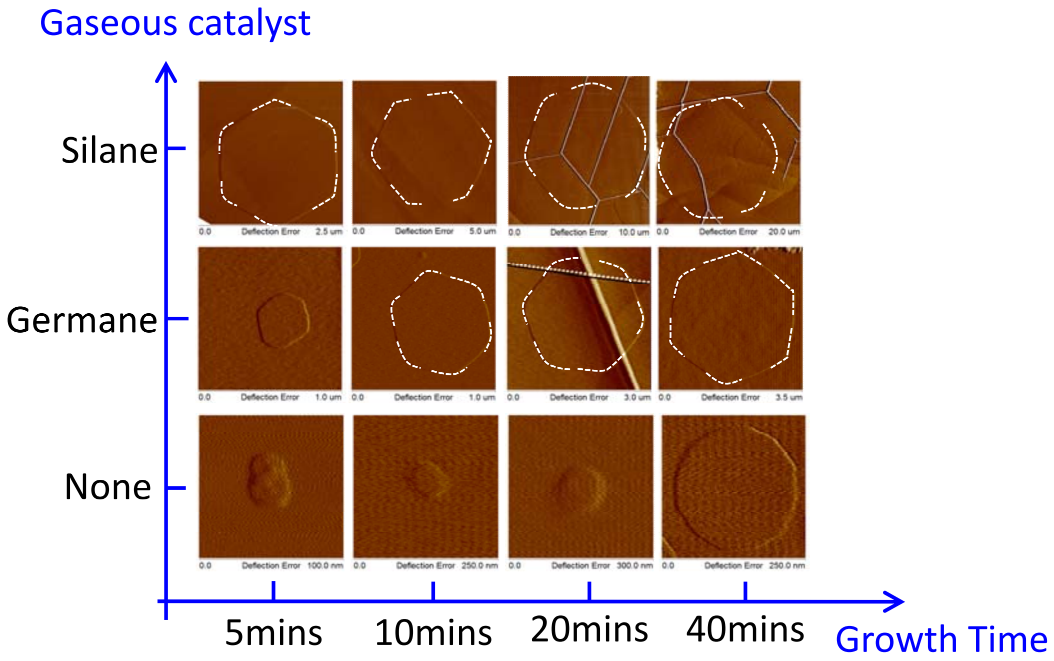

Supplementary Figure 2 | Determining the growth rate of single crystalline graphene domain. The size of single crystalline graphene is plotted as the function of the growth time in the presence of different gaseous catalysts. The dashed lines frame the shape of the graphene domains. All sizes of graphene domain are represented by the diagonal length of hexagonal graphene crystal. The growth temperature was kept at 1280□.

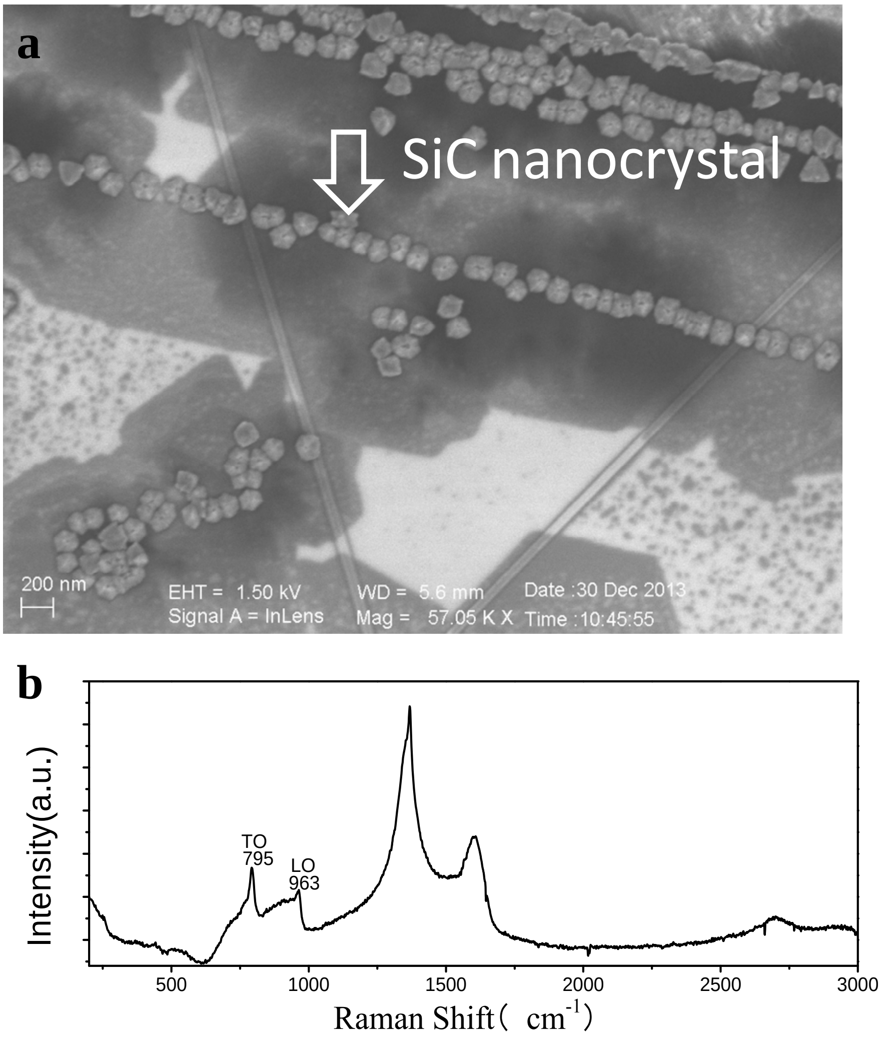

Supplementary Figure 3 | SiC formation in high silane flow rate. Characterization of the graphene materials grown for 10 minutes in the condition No.9 of Supplementary Table 1. (a) The image of scanning electron microscopy (SEM) on *h*-BN and (b) Raman spectrum of the graphene in *h*-BN. The SEM image shows that there are nano-crystals on *h*-BN, and the Raman characterization indicates the nano-crystals are mostly made from SiC.[1]

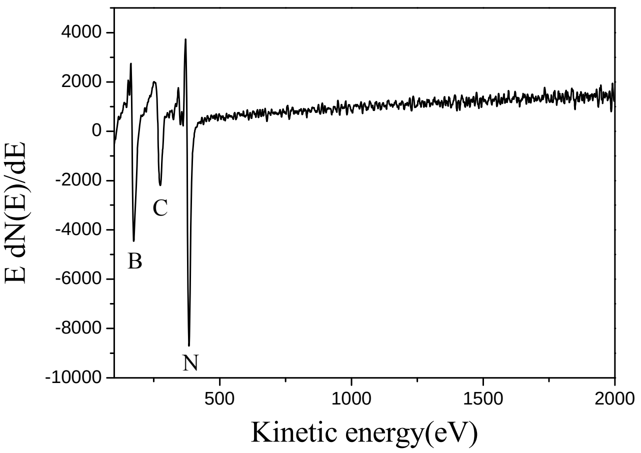

Supplementary Figure 4 | Auger spectrum of graphene/*h*-BN (in an area of 10×10μm) in derivative mode plotted as a function of energy. Different peaks for B, C and N are apparent. The survey spectrum shows the presence of boron, carbon and nitrogen. No obvious signal of Si/Ge is detected within the detection limit of the AES. These results indicate that the graphene grown on *h*-BN is almost Si/Ge-free.

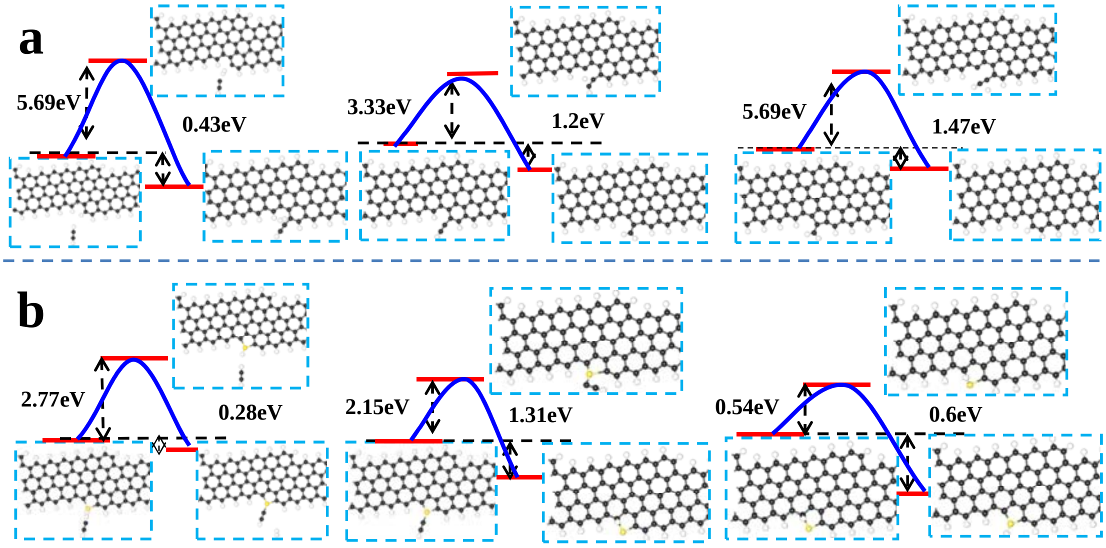

Supplementary Figure 5 | The molecular dynamic simulation of the growth behaviors at the hydrogen terminated zigzag edge of graphene domains. The repeatable cycles of incorporating a C_2H_2 molecule onto (a) H-terminated and (b) H- and Si- terminated graphene edges. Black, white and yellow balls represent carbon, hydrogen and silicon atom, separately.

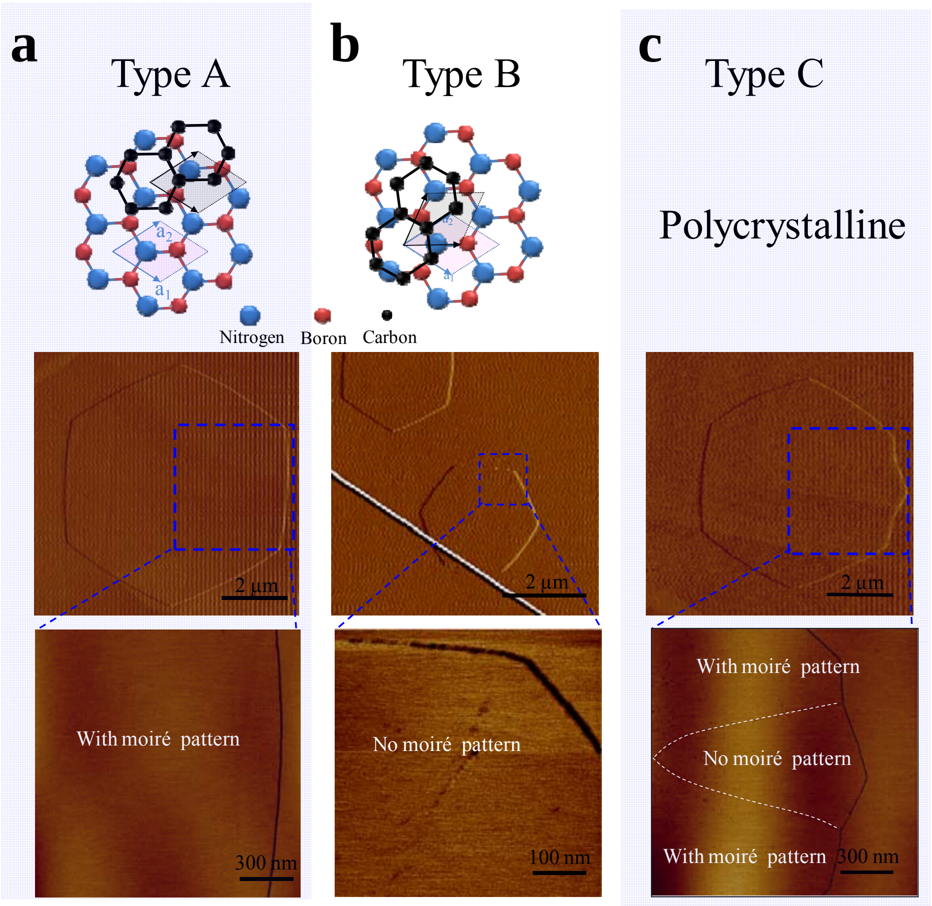

Supplementary Figure 6 | Classification of graphene domains grown on the surface of *h*-BN. The topography of the "different type" graphene domains on *h*-BN surface were measured. (a) Type "A" domain is precisely aligned with that of the underlying *h*-BN, and shows moiré pattern with the periodicity of about 13.9 nm; (b) Type "B" domain shows regular hexagonal shape but the graphene lattice is rotated about 30° relative to the underlying *h*-BN lattice; (c) Type "C" shows typical polycrystalline structure with moiré pattern detectable on some sub- domains but not on the others.

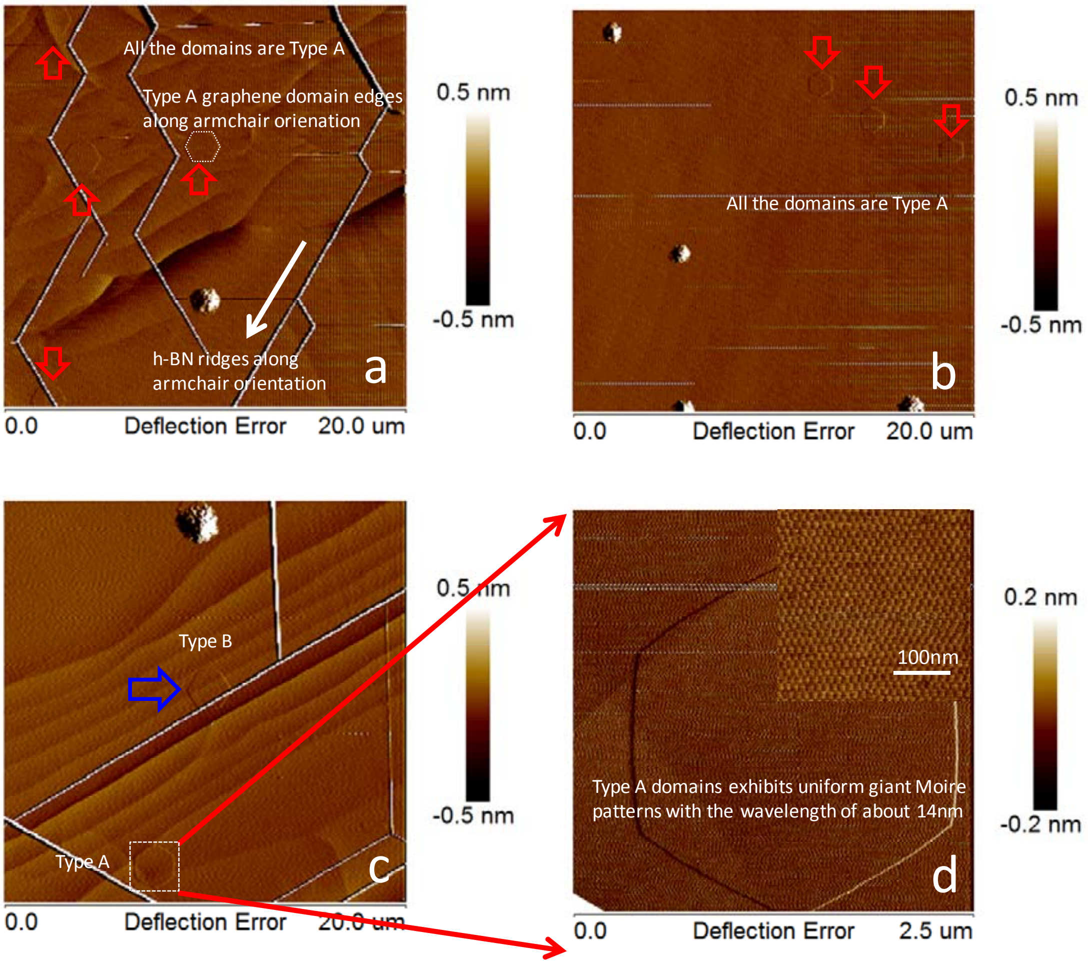

Supplementary Figure 7 | Typical AFM images for graphene domains. (a) & (b) typical AFM images for the "Type" survey, the domains are all "Type A" single crystalline graphene domain. The ridges are always used for the determination of the domain type; (c) The AFM image showing a "Type A" domain and a "Type B", where "Type A" exhibits uniform giant moiré patterns with a periodicity of 13.9 nm can be clearly seen (d).

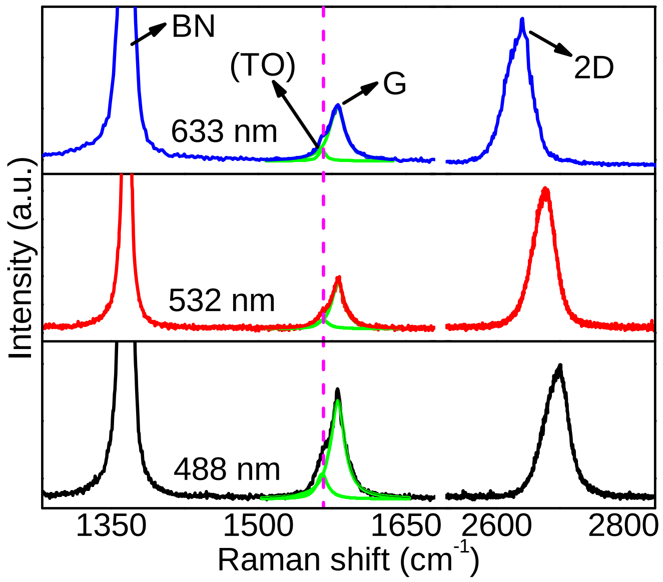

Supplementary Figure 8 | Exciting energy dependence of the Raman spectrum of precisely aligned graphene. Raman spectra were obtained with a WITec micro-Raman instrument possessing excitation laser lines of 488/532/633 nm. An objective lens of 100× magnification and a 0.95 numerical aperture (NA) was used, producing a laser spot that was ~0.5 μm in diameter. The laser power was kept less than 1 mW on the sample surface to avoid laser-induced heating. The excitation-laser-energy-dependent-Raman spectroscopy was used to support the conclusion that the shoulder peak located at 1565 cm^{-1} in precisely aligned graphene domain is a TO phonon originated from the inter-valley umklapp scattering activated by graphene/*h*-BN super-lattice.

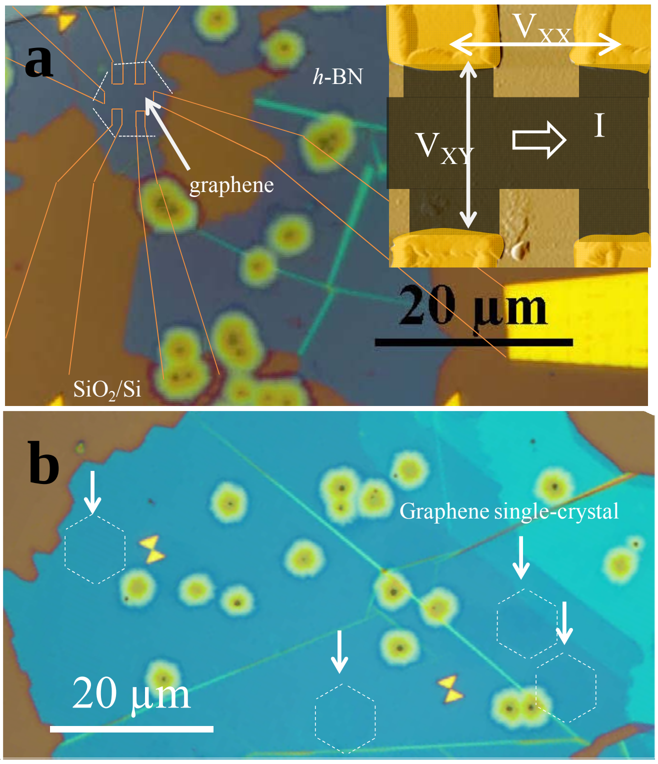

Supplementary Figure 9 | Images of graphene domain and device design. (a) Device design for a single crystal graphene domain grown on *h*-BN obtained by GVC-CVD, the inset shows a false-colored AFM image of a device on *h*-BN fabricated from the graphene single crystal grown on *h*-BN; (b) Optical image of a *h*-BN flake, the dashed white line marks the edges of single crystal graphene domains on *h*-BN.

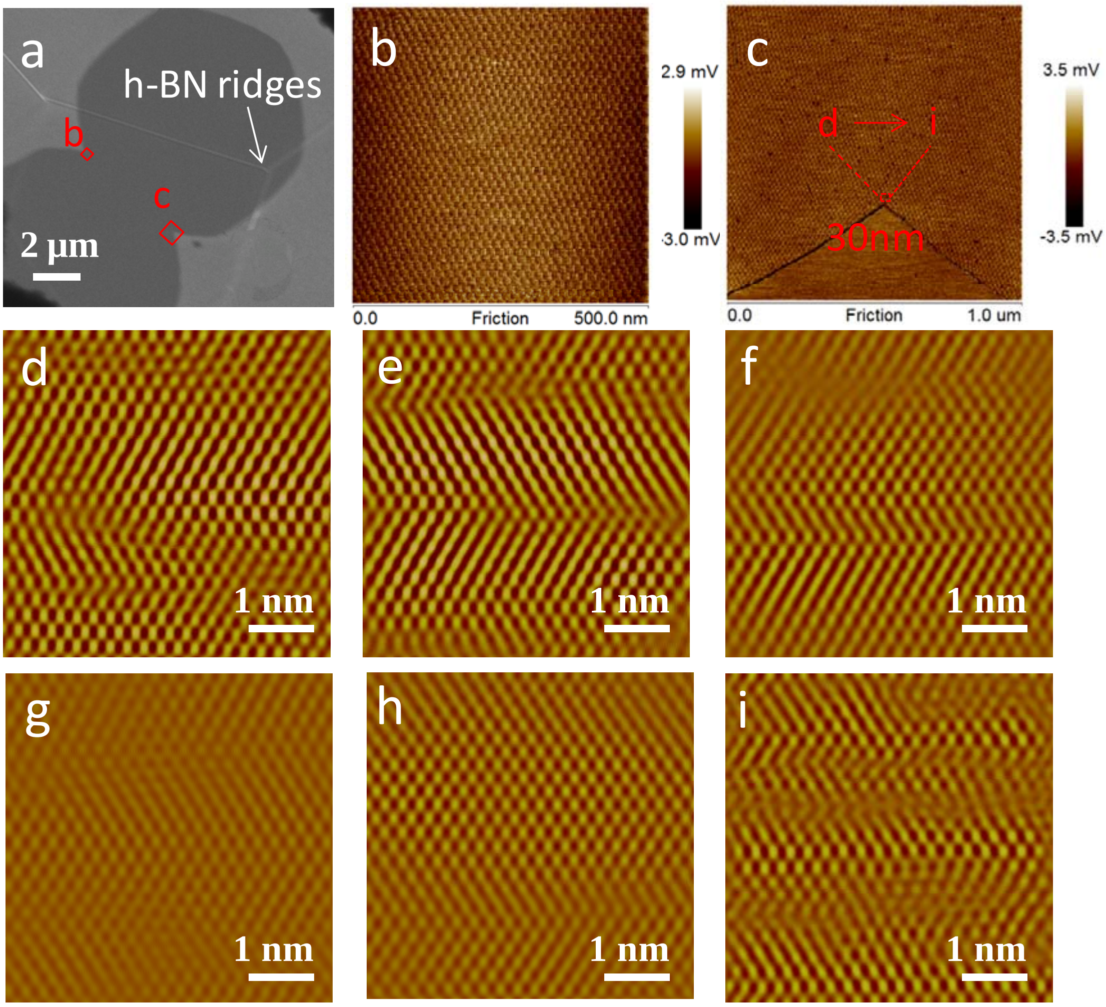

Supplementary Figure 10 | Investigation on domain boundary where adjacent domains are coalescing. (a) A SEM image of adjacent graphene domains on *h*-BN. Red boxes indicates the location where adjacent domains are merging. (b) and (c) AFM images of the boundary area within the red boxes of panel (a). The AFM images shows uniform moiré patterns distribution on the graphene domain. The moiré patterns exhibit good continuity. (d), (e), (f), (g), (h) and (i) are atomic scans ($5 \times 5nm^2$) taken from red box (30×5 nm^2) in panel (c) (spatially continuous from left to right). These atomic images show the evolution of graphene domains to uniform monolayer during the CVD growth. By careful examination of atomic resolution AFM, no obvious domain boundary was found. These AFM images clearly show adjacent graphene domains have same lattice orientation and coalesce without grain boundary defects. It is confirmed that the adjacent domains merged at the boundary seamlessly.

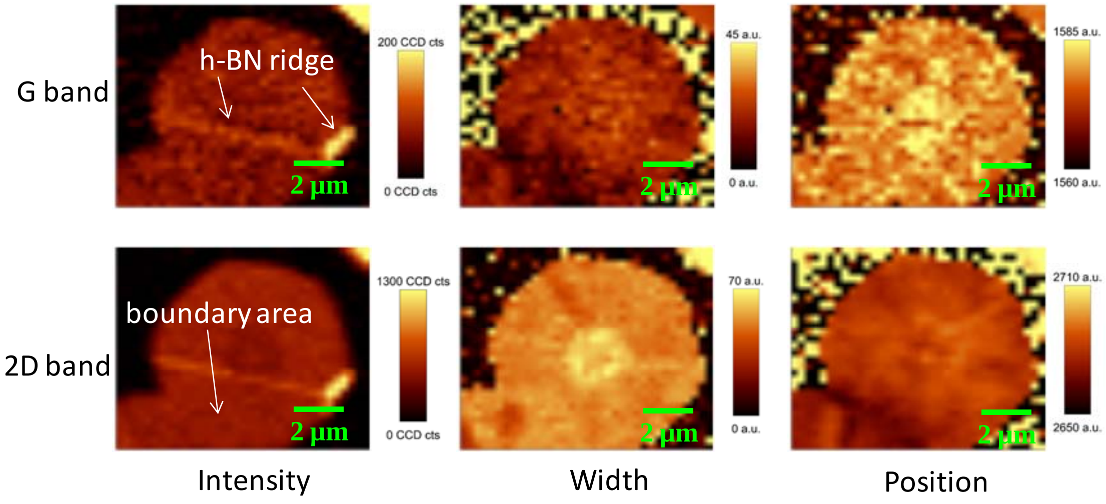

Supplementary Figure 11 | Raman G-band and 2D-band high-resolution-mapping of the grain boundary between two precisely aligned graphene domains. The high resolution Raman images show no obvious sign of grain boundary defects in the merged area. It is found that there is a very small red shift of 2D position in the merging area between domains. Other area is very uniform. The small shift of 2D peak (corresponding to < 0.1% strain) may attribute to the small stain introduced during the coalescing of graphene domains.

Supplementary Tables

	Ar with 5%SiH_4 (sccm)	C_2H_2 (sccm)	Pressure (Pa)	Temperature (° C)	Duration (mins)	Graphene Diameter (μm)	Growth rate (μm/min)	Any SiC observed?
No.1	2	4	5	900	60	2.5	0.042	No
No.2	4	4	5	900	60	2.6	0.043	No
No.3	8	4	5	900	60	2.6	0.043	No
No.4	16	4	5	900	60	2.8	0.047	No
No.5	2	4	5	1280	20	4	0.2	No
No.6	4	4	5	1280	20	6.8	0.34	No
No.7	8	4	5	1280	20	7.5	0.375	No
No.8	16	4	5	1280	20	8.2	0.41	No
No.9	32	4	5	1280	20	8	0.4	Yes
No.10	2	4	5	1350	10	10	1	No
No.11	4	4	5	1350	10	9.8	0.98	No
No.12	8	4	5	1350	10	9.5	0.95	No
No.13	16	4	5	1350	10	9.1	0.91	Yes

Supplementary Table 1 | Summary of CVD conditions evaluated for graphene growth on *h*-BN using gaseous catalyst.

	G band		(TO) band		2D band	
Excitation Laser energy	Position (cm^{-1})	FWHM (cm^{-1})	Position (cm^{-1})	FWHM (cm^{-1})	Position (cm^{-1})	FWHM (cm^{-1})
633 nm	1580	18	1565	12	2635.6	49
532 nm	1580.9	15	1565.8	13.7	2675.3	40
488 nm	1580.6	17	1565	13	2695	44

Supplementary Table 2 | Summary of peak position and full width at half maximum (FWHM) of the Raman peaks shown in Supplementary Fig. 8.

Year	Temp.	Mobility (cm^2/Vs)	Substrate	Geometry	Method	Growth rate	comments	Ref.
2008	240K<br>5K	120,000<br>170,000	None	Hall and FET	Mechanically exfoliated from bulk graphite	N.A.	Freestanding graphene	2
2009	1.6K	3,750	SiO_2	FET	CVD on Cu	N.A.	Etching and Transfer needed	3
2009	Room Temp. (RT)	4,050	SiO_2 and Al_2O_3	dual-gated FETs	Thermal CVD on copper	3~5µm/mi n	Etching and Transfer needed	4
2010	RT	800-16,000	SiO_2	FETs	Thermal CVD on copper	3~10 µm/min	Etching and transfer needed	5
2010	2K	25,000<br>25,000-140,000	*h*-BN	Hall mobility<br>FET	Mechanically exfoliated from bulk graphite	N.A.	Transfer needed	6
2011	RT	4,000	SiO_2	FETs	Thermal CVD	0.1~4 µm/min	Etching and Transfer needed	7
2011	RT<br>2K	3,000<br>10,500	Al_2O_3	Hall mobility	Thermal CVD	N.A.	Transfer free	8
2011	RT	1800	SiC	FET	Epitaxy and CVD	N.A.	Transfer free	9
2011	4.2K	9,200–28,800<br>4000–5400	*h*-BN<br>SiO_2	Hall bar	CVD on Cu	3~10µm/ min	Etching and Transfer needed	10
2011	RT	<1000~10,000	SiO_2	FET	Thermal CVD on Cu	1µm/min	Etching and transfer needed	11
2012	RT	1,000–2,500	SiO_2	FET	Thermal CVD on Cu	3.15µm/m in	Etching and transfer needed	12
2012	RT	7,100	SiO_2	FET	Thermal CVD on Pt	4.17µm/m in	Etching and transfer needed	13
2012	1.6K	27,200~44,90 0	*h*-BN	Hall bar	Thermal CVD on Cu	0.67~2.1µ n/min	Etching and transfer need	14
2012	RT	277± 91(e)<br>227 ± 66(h)	Al_2O_3	FET	Thermal CVD	2.78nm/m in	Transfer free	15
2012	RT	2,000	Al_2O_3	Hall mobility	Thermal CVD	50nm/min	Transfer free	16
2013	RT	1,300-5,650	$Si_3N_4/SiO_2/Si$	FET	Oxygen-Aided CVD	2.5nm/mi n	Transfer free and polycrystalline	17
2013	1.5K	5,000	*h*-BN	FET	Plasma-enhanced CVD	0.65nm/m in	Transfer free and well aligned	18
2013	Low T	20,000-80,000	*h*-BN	FET and Hall	Mechanically exfoliated from bulk graphite	N.A.	Transfer needed	19
2013	Low T	10,000~100,0 00	*h*-BN	FET and Hall	Mechanically exfoliated from bulk graphite	N.A.	Transfer needed	20
2014	RT	531	SiO_2	FET	CVD on	1.66nm/m in	Transfer free	21
2014 (this work)	RT	~17,000<br>19,000(e)-23,000(h)	*h*-BN	FET and Hall	Gaseous Catalyst Assisted-CVD	1µm/min	Transfer free and well aligned	this work

Supplementary Table 3 | Survey on mobility of charge carriers in graphene reported in literatures. The properties of CVD graphene samples, especially the electronic performance, are highly dependent on factors such as synthesis and processing techniques, and adjacent materials. The survey in carrier mobility shown here indicates that the mobility prefers the flat *h*-BN with inert surface.

Supplementary Notes

Supplementary Note 1 | the effect of solid catalyst

To qualitatively understand the role of silicon (germanium) atom in the growth of graphene, solid silicon, solid germanium and their alloy were placed near *h*-BN flakes, respectively. The Ge/Si alloy consists of 30% Si in molar ratio. We found that the presence of solid silicon/germanium/their alloy can effectively improve growth rate of graphene when heating them to 1280□ or higher. It indicates that silicon/germanium vapor plays a role of catalyst in graphene growth on *h*-BN. The results illuminate us that the saline/germane may be effective catalysts for graphene growth on *h*-BN.

Supplementary Note 2 | Key parameters for the graphene growth

The graphene growth was influenced mainly by the gaseous catalyst and growth temperature:

1. Argon/silane mixture flow rate: a C_2H_2 flow and a mixture of silane/argon (the mole ratio of silane to argon was 5%) were introduced into the system for the graphene growth. High flow rate of the argon/silane mixture resulted in a rapid growth rate. Some growth results with different argon/silane mixture flow rate are summarized in Supplementary Table 1.
2. Growth temperature: Temperature plays an important role in decomposition of hydrocarbons and activates the catalyst. An increase in the growth temperature provides the possibility for increasing the growth rate. Some growth results with different growth temperature are summarized in Supplementary Table 1. The quality of the graphene grown under different temperature shows little difference via Raman measurement.
3. Too much silane could lead to the formation of SiC as shown in Supplementary Fig. 3.

Supplementary Note 3 | C_2H_2 as a precursor.

It is worthy to note that the above picture hold valid only when C_2H_2 is used as a precursor. If we use CH_4 as a carbon precursor, the atmospheric composition in the reaction chamber will be very different. We experimentally verified that the catalytic effect is not obvious, if using CH_4. The catalytic effects due to silicon or germanium

atoms are further validated by heating solid silicon or Si/Ge alloys to different temperatures at different pressures, thus producing different vapor pressures of Si and Ge atoms.

Supplementary Discussion

Simulation of the growth behaviors at zigzag edges.

To get a more detailed understanding on the growth mechanism at zigzag edges, we performed a DFT calculation and the results are shown in Supplementary Fig. 5. We assumed that the growth frontier is passivated by hydrogen atoms, and the C_2H_2 molecule is the carbon feedstock. In the non-catalyst situation (Supplementary Fig. 5a), the C_2H_2 molecules need three steps to incorporate to the graphene edge to form a new carbon ring. The energy barriers for the three steps are 5.69eV, 3.33eV and 5.69eV, respectively. After the new carbon ring is formed, the growth frontier steps forward and form a same structure as that before the C_2H_2 molecule integrated in the carbon ring. In the silicon catalyst situation (Supplementary Fig. 5b), we start with one silicon atom being absorbed on the growth frontier, in the growth circle such silicon atom serves as a bridge between carbon dimer and graphene, which lowers the energy needed for dehydrogenation of C_2H_2 and formation of carbon-carbon bonds to 2.77eV, 2.15eV and 0.5eV. The much lowered energy barriers account for the greatly enhanced growth rate.

Classification of graphene domains grown on the surface of *h*-BN.

Actually, we scanned the surface of *h*-BN flakes and measured the topography of the “different type” graphene domains on *h*-BN surface by AFM. We measured about 200 pieces of *h*-BN flakes for the statistics in Fig. 3. In total, about 1600 graphene domains are measured. There are some advices in such an investigation. 1) The edges orientation of graphene domain, moiré patterns and the atomic resolution images of graphene on *h*-BN help to determine the type of graphene domains. The details about the investigating methods are introduced in our earlier publication (S. Tang et al., Precisely aligned graphene grown on hexagonal boron nitride by catalyst free chemical vapor deposition. Sci. Rep. 3, 2666 (2013)). 2) There are always ridges formed on the surface of *h*-BN. The

h-BN ridges are caused by the different thermal expansion coefficients between *h*-BN and underling SiO_2 surface. We found that almost all the ridges are precisely along the armchair direction of *h*-BN. As most of the graphene domains are of edges in parallel with armchair direction, one could make use of the ridges to know the lattice orientation of *h*-BN. It really can save much time. 3) Graphene single crystalline domains always exhibit a hexagonal shape. The domains show a giant superlattice with wavelength of about 14 nm if the domains are precisely aligned with the *h*-BN. 4) the superlattices are also known as “moiré patterns”, and high quality graphene domains have very uniform moiré patterns.

Although the investigations are actually tedious and time-consuming, it is worthy to do such an investigation to know the uniformity of the graphene domain. Supplementary Fig. 7 gives more examples for the explanation of the type survey.

Transport measurement

Electronic transport measurements in a Hall bar configuration were also carried out to characterize the graphene single crystal grown on *h*-BN. The gate voltage (V_g) dependence of the longitudinal resistance (R_{xx}) at different temperature is plotted in Fig. 5a. The main peak in R_{xx} at V_g = -5 V, represents graphene’s main neutrality point. Two satellite peaks symmetrically appear on both sides of the main neutrality point. As the temperature decreases, these two satellite peaks, become more obvious. The satellite peak on the hole side appears much stronger than that on the electron side. The satellite peaks in transport properties are believed to be related to the spectral reconstruction in graphene brought to contact with *h*-BN. The superlattice potential induced by the *h*-BN results in the appearance of secondary Dirac points (SDP) in graphene’s energy dispersion. They can be observed as the satellite peaks in the transport measurement after the Fermi energy is tuned to reach the reconstructed part of the spectrum. The resistances (R) at the Dirac Point (DP) and satellite peak at the hole branch as a function of T are plotted in the inset of Fig.5a, both of them exhibit very weak temperature dependence, the results are different from earlier reports. One possible reason is that the commensurate state is suppressed.[24]

As the satellite resistance peaks in the transport data originates from the moiré pattern, the wavelength of moiré pattern can be estimated by measuring the relative position of the satellite resistance peaks. As the energy separation between the DP and the secondary Dirac point (SDP) is $E_{SDP} = \frac{h v_F}{\sqrt{3}\lambda}$, where λ is superlattice period, v_F is graphene's Fermi velocity and h is Planck's constant. The Fermi level can be tuned by applying the external gate voltage: $E_F = \frac{h}{2\pi} v_F \sqrt{\pi C_g (V_g - V_{NP})/e}$, where "$e$" represents elementary charge, the effective capacitance C_g can be estimated at about 10.5 nF·cm^{-2}. Thus, we can roughly estimate the wavelength of the morie pattern from the equation $\lambda = 2\sqrt{\frac{\pi e}{3C_g(V_g - V_{NP})}}$, where the V_g represents gate voltage, V_{NP} represents gate voltage at neutral point. Given that V_g-V_{NP} = 33 V and C_g≈10.5nF/μ m^2, we can get $\lambda \approx$ 14.1 nm . The wavelength of the morie pattern derived from the gate voltage at which the secondary Dirac point occur matches well with our results obtained from AFM measurement.[25,26]. From Fig. 5a, the electrical field mobility at 300 K is about 17,000 cm^{2}V^{-1}s^{-1}, which is extracted from a density independent mobility model $R_{total} = \frac{L/W}{\mu\sqrt{(n_0 e)^2 + (V_g - V_{dirac})^2 C_g^2}}$, where n_0 is the residual carrier density induced by charge impurities.[27] L and W represent channel length and width, respectively. Both of them equals to 1 μm in this device. Here, the dielectric constant of h-BN is comparable with that of SiO_2. Magnetotransport for the graphene/h-BN hetero-structure is also measured at T ≈ 300 K. Longitudinal resistance (R_{xx}) and Hall resistance (R_{xy}) as a function of gate voltage taken in a magnetic field of B = 9 T are shown in Fig. 5b. The extracted Hall mobility $\mu = \frac{R_{xy} \cdot L}{R_{xx} \cdot W} \cdot \frac{1}{B}$ is about 19,000 cm^{2}V^{-1}s^{-1} for the holes and ~23,000 cm^2/V·s for electrons.

The color plots of the R_{xx} and R_{xy} as a function of both gate voltage and magnetic field are shown in Fig.5c and 5d, respectively. The standard quantum Hall effect (QHE) for graphene, is observed with valleys in R_{xx} (Fig.5c) and plateau in R_{xy} (Fig. 5d) at filling factors $\nu = \pm 4(n + 1/2) = \pm 2, \pm 6, \pm 10$ *and* $\pm 14,...$ where $n = 0, 1...$ is the LL index. The

features are the characteristic of the gapless Dirac spectrum of graphene. Similar features fan out from secondary Dirac point in the precisely aligned graphene on *h*-BN and the Dirac Fermion physics near the main Dirac point is unperturbed. It is noted that the resistance peak of the SDP on the hole doping regime broadens with the increase of magnetic field. Over all, the half integer QHE observed in the precisely aligned graphene on *h*-BN indicates the high quality of precisely aligned graphene grown on *h*-BN.

Supplementary References

[1] S. Nakashima and H. Harima, Raman Investigation of SiC Polytypes, *phys. stat. sol. (a)* 162, 39 (1997).

[2] K. I. Bolotin, K. J. Sikes, J. Hone, H. L. Stormer, P. Kim, Temperature-Dependent Transport in Suspended Graphene. *Phys. Rev. Lett.* **101**, 096802 (2008).

[3] K. S. Kim, *et al.* Large-scale pattern growth of graphene films for stretchable transparent electrodes. *Nature* **457**, 706-710 (2009).

[4] X. Li, *et al.* Large-Area Synthesis of High-Quality and Uniform Graphene Films on Copper Foils. *Science.* **324**, 1312-1314 (2009).

[5] X. Li, *et al*. Graphene Films with Large Domain Size by a Two-Step Chemical Vapor Deposition Process. *Nano Letters.* **10**, 4328-4334 (2010).

[6] C. R. Dean, et al. Boron nitride substrates for high-quality graphene electronics. *Nat. Nano.* **5**, 722-726 (2010).

[7] X. Li, *et al*. Large-Area Graphene Single Crystals Grown by Low-Pressure Chemical Vapor Deposition of Methane on Copper. *Journal of the American Chemical Society.* **133**, 2816-2819 (2011).

[8] M. A. Fanton, *et al.* Characterization of Graphene Films and Transistors Grown on Sapphire by Metal-Free Chemical Vapor Deposition. *ACS Nano.* **5**, 8062-8069 (2011).

[9] W. Strupinski, *et al*. Graphene Epitaxy by Chemical Vapor Deposition on SiC. *Nano Letters.* **11**, 1786-1791 (2011).

[10] W. Gannett, *et al.* Boron nitride substrates for high mobility chemical vapor deposited graphene. *Appl. Phys. Lett.* **98**, 242105 (2011).

[11] Q. Yu, *et al.* Control and characterization of individual grains and grain boundaries in graphene grown by chemical vapour deposition. *Nat Mater.* **10**, 443-449 (2011).

[12] D. Geng, *et al.* Uniform hexagonal graphene flakes and films grown on liquid copper surface, *PNAS.* **109**, 7992-7996 (2012).

[13] L. Gao, *et al.* Repeated growth and bubbling transfer of graphene with millimetre-size single-crystal grains using platinum. *Nat. Commun.* **3**, 699 (2012).

[14] N. Petrone, *et al.* Chemical Vapor Deposition-Derived Graphene with Electrical Performance of Exfoliated Graphene. *Nano Lett.* **12**, 2751-2756 (2012).

[15] H. J. Song, *et al*. Large scale metal-free synthesis of graphene on sapphire and transfer-free device fabrication. *Nanoscale.* **4**, 3050-3054 (2012).

[16] J. Hwang, et al. van der Waals Epitaxial Growth of Graphene on Sapphire by Chemical Vapor Deposition without a Metal Catalyst. *ACS Nano.* **7**, 385-395 (2012).

[17] J. Chen, *et al.* Oxygen-Aided Synthesis of Polycrystalline Graphene on Silicon Dioxide Substrates. *Journal of the American Chemical Society.* **133**, 17548-17551 (2011).

[18] W. Yang, *et al*. Epitaxial growth of single-domain graphene on hexagonal boron nitride. *Nat. Mater.* **12**, 792-797 (2013).

[19] L. A. Ponomarenko, *et al*. Cloning of Dirac fermions in graphene super-lattices. *Nature* **497**, 594-597 (2013).

[20] X. Li, *et al.* Graphene Films with Large Domain Size by a Two-Step Chemical Vapor Deposition Process. *Nano Lett.* **10**, 4328-4334 (2010).

[21] J. Chen, et al. Near-Equilibrium Chemical Vapor Deposition of High-Quality Single-Crystal Graphene Directly on Various Dielectric Substrates. *Adv. Mater.* **26**, 1348-1353, (2014).
